# In Situ Wireless Channel Visualization Using Augmented Reality and Ray Tracing

**DOI:** 10.3390/s20030690

**Published:** 2020-01-27

**Authors:** George Koutitas, Varun Kumar Siddaraju, Vangelis Metsis

**Affiliations:** 1 Electrical Engineering, Texas State University, San Marcos, TX 78666, USA; varunsiddaraju@gmail.com; 2 Computer Science, Texas State University, San Marcos, TX 78666, USA; vmetsis@txstate.edu

**Keywords:** 5G networks, augmented reality, ray tracing, spatial mapping, network signal visualization

## Abstract

This article presents a novel methodology for predicting wireless signal propagation using ray-tracing algorithms, and visualizing signal variations in situ by leveraging Augmented Reality (AR) tools. The proposed system performs a special type of spatial mapping, capable of converting a scanned indoor environment to a vector facet model. A ray-tracing algorithm uses the facet model for wireless signal predictions. Finally, an AR application overlays the signal strength predictions on the physical space in the form of holograms. Although some indoor reconstruction models have already been developed, this paper proposes an image to a facet algorithm for indoor reconstruction and compares its performance with existing AR algorithms, such as spatial understanding that are modified to create the required facet models. In addition, the paper orchestrates AR and ray-tracing techniques to provide an in situ network visualization interface. It is shown that the accuracy of the derived facet models is acceptable, and the overall signal predictions are not significantly affected by any potential inaccuracies of the indoor reconstruction. With the expected increase of densely deployed indoor 5G networks, it is believed that these types of AR applications for network visualization will play a key role in the successful planning of 5G networks.

## 1. Introduction

The forthcoming 5G network technology is expected to create new challenges in terms of indoor network planning [1]. One of the most important characteristics of 5G networks is the need to densely deploy the radio access in order to provide high bandwidth coverage at extremely high-frequency bands. It is clear that there is a need for accurate and easy-to-use indoor network planning technologies. For commercialization purposes, these technologies should not only be used by experts in the field of Radio Frequency (RF) design, but also from the end-users that require to deploy 5G access points in their premises.

One of the most complex processes for indoor channel prediction is the computation of the vector model of the indoor environment. This has the form of a facet model where doors, windows, and walls are described as a structure of corner coordinates in the 3D space [2]. 5G networks are using high-frequency bands such as 6 GHz or above 24 GHz, and the effect of indoor clutter is significant [3]. Thus, the accuracy of the modeling of the indoor environment becomes a significant component of the overall channel prediction process. Usually, the facet model is taken from the architectural cad designs of the apartment, and complex conversions need to take place. Unfortunately, in many cases, the architectural design is not available, and modeling of the vector maps requires extensive measurements of the interior space. It is obvious that those processes are complex, expensive, and require the involvement of specialized third parties. 

Recent advances in Augmented Reality (AR), Artificial Intelligence (AI) techniques have created new opportunities for spatial mapping, spatial understanding and visualization [4]. Spatial mapping is considered one of the most fundamental components of AR since it allows the application to recognize the scanned space. With this feature, holograms can interact with the physical environment since the information of the physical dimension of obstacles is known [5]. Existing spatial mapping algorithms only provide a measure of the physical dimension of obstacles neglecting the vector format (facet model) that is required by the ray-tracing prediction algorithm. In addition, new AR applications have been published that visualize signal strength [6]. The application in [6] records the Received Signal Strength Indicator (RSSI) of a smartphone device or from the API of Magic Leap headset and uses a spatial anchor to deploy the RSSI in space. The user is able to visualize the signal power in the environment using the AR headset. It should be noted that Magic Leap has not published the implementation details of how they have implemented their network visualization and thus cannot be replicated by other researchers. 

This article integrates AR, spatial mapping and ray tracing channel prediction algorithms in one platform to empower users with the ability to visualize the predicted wireless channel in real-time in the form of holographic representations. 

The novelty of the paper can be summarized as:

- We developed and investigated the performance of three spatial mapping algorithms that converts image files or 3D objects to indoor vector maps, called the facet model. The facet model is required for the ray tracing algorithm to perform signal strength predictions. The first solution called the image to facet algorithm does not require the use of expensive AR devices, and results can be obtained by using the camera of a typical smartphone device. The algorithm converts the .jpeg images of the walls of a room to a vector facet model. The importance of this solution is that it is low-cost and can allow any smartphone device to host the proposed application. The second algorithm is called min-max spatial mapping and converts an object file of vertices derived by an AR device to the indoor facet map. This algorithm requires the use of a depth camera, which is usually part of expensive AR devices. Furthermore, the spatial understanding algorithm uses an existing Software Development Kit (SDK) to extract the walls of the room and create the facet model.
- In addition, we developed a channel prediction ray tracing algorithm, based on the ray launching technique, and integrated it into the AR engine to create an output suitable for the holographic presentation. The channel prediction algorithm considers any combination of propagation mechanisms such as multiple reflections and transmission through walls as well as single diffractions from wall corners.
- We also evaluated the proposed solutions over a residential apartment and showed that the proposed 2D image-to-facet model provides accurate results and can be hosted on any smartphone device.
- Compared to existing applications that visualize field strength, the proposed solution goes multiple steps beyond because it does not require measurements since it estimates the radio signals making it suitable for radio planning. In addition, it considers the indoor environment in the field predictions, whereas other apps simply record the signal strength.

Finally, the paper integrated the developed technologies in one “beta” application that empowers the user with the ability to walk inside an indoor environment and visualize in situ radio coverage.

## 2. System Model of Augmented-Rays

AR is a technology that connects the real physical world with the virtual world. This is performed by advanced visualization techniques that overlay holograms on top of the physical space. In many cases, AR is also called Mixed Reality (MR), where holograms can interact with the physical world using the concept of occlusion. One of the enablers of AR/MR applications is the technology called spatial mapping [5,7]. Spatial mapping creates a detailed representation of real-world surfaces of the environment so as holograms can interact with the physical environment. Spatial mapping uses sophisticated image processing and AI algorithms, and it may require the use of depth cameras [8]. 

**Figure 1:**
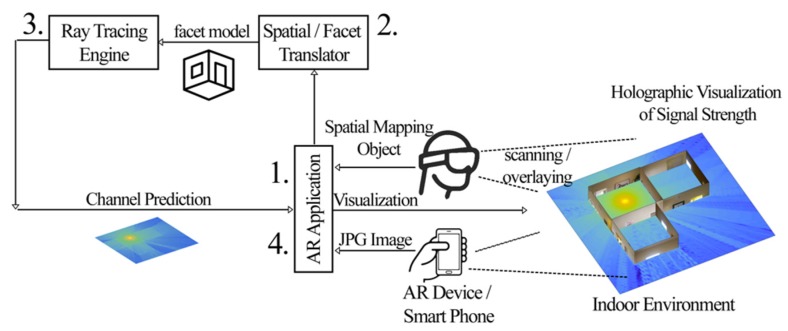
The system architecture of the proposed network visualization technology.

The proposed system model is presented in Figure 1, where the sequence of the steps is labeled with numbers. The system can be applied in two types of devices such as an AR headset called *Hololens* or a typical smartphone device with a camera, without AR and depth camera functionalities. At the first step of the overall process, the user is required to scan the environment of interest. When the user has an AR device, the scanning process creates an object file, representing the surface mesh of the obstacles. When the user uses a smartphone camera, the scanning process captures the set of images, each one representing a wall of the indoor environment. 

The second step involves the spatial mapping-to-facet translation engine where the scanned surfaces are converted to a facet model. The translation engine is a CPU unit that can be hosted within the device or in the Cloud. During the translation process, the image or object files are processed and converted to a vector format that is used by the ray tracing algorithm. The facet model describes each wall, door, or window of the indoor environment as a set of corner points together with the constitutive parameters of the material [2,9]. 

The third step of the process is the ray tracing engine, which is responsible for predicting the radio propagation and wireless channel based on the geometry of the indoor clutter. The predictions are performed by a ray tracing algorithm, based on the ray launching technique, which uses principles of Geometric Optics (GO) and Uniform Theory of Diffraction (UTD). The output of the ray tracing is a file that contains information about the strength of every location of the indoor environment. This is usually described as a mesh-grid that spans the entire indoor space.

Finally, the AR device receives the output of the ray tracing engine and overlays the colormap representation of the channel prediction on the physical environment in the form of a holographic representation. An occlusion technique was used to create a user experience User Experience (UX) based on MR. 

## 3. Spatial Mapping and Facet Model Translation Engine

Spatial mapping creates a 3D reconstruction of the environment. This is usually performed by depth cameras that scan the environment of interest. Characteristic examples of such cameras are the Red Green Blue (RGB) depth (RGB-D) [8,10] and monocular cameras used for Simultaneously Localization and Mapping (SLAM) [11,12]. The existing spatial mapping algorithms create a mesh representation of the space that cannot be directly applied in ray tracing channel estimation techniques. This is because the indoor environment should be represented in a vector format called the facet model. This paper proposes three different algorithms that can create a facet model of an indoor environment based on inputs from smartphone cameras or the Hololens AR device [5]. 

### 3.1. Image to Facet Model Algorithm

In the most simple and inexpensive version, the user is required to “scan” the indoor environment by capturing the images of each interior wall of the room using a typical smartphone device. The algorithm processes the images, identifies the walls and doors, computes their corner coordinates, and reconstructs the vector-facet map. The procedure used for identifying a wall and its dimensions is described in Algorithm 1. The algorithm takes as input a captured image, where all four corners of a wall are within the field of view of the camera, but possibly occluded, and the height of the wall, which we assume to be a constant parameter provided by the user. If the field of view of the camera is not wide enough to capture the entire wall into one frame, the built-in Panorama mode can be used to capture a wider field of view.
**Algorithm 1.** Wall Detection1: **procedure** Detect_wall(image, wall_height)2:  Find Edge pixels using *Canny Edge Detector*.3:  Find (top, bottom, left, right)-most straight lines on the edge image using *Hough Transform*.4:  Compute *intersecting points* of lines. Consider those as the four *corners* of the wall.5:  Apply corner detection to detect *corners* in the image and find the best candidates for corner locations by comparing with line intersection points.6:  Use user input of *wall_height* and detected wall corners to estimate the length of horizontal lines and overall wall *dimensions*.7: **return** wall dimensions8: **end procedure**

**Figure 2:**
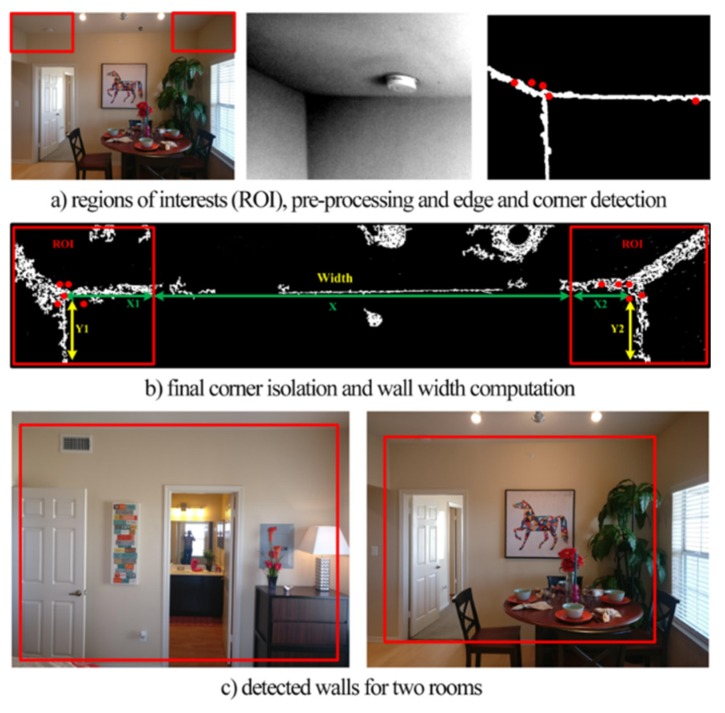
The 2D image-to-facet translation processes.

The first step applied to the input image is the pre-processing phase where the image is converted into grayscale and also automatically cropped in the Regions of Interest (ROI), which are the top and left corners of the wall, as shown in Figure 2a. This eliminates potential errors on the edge detection step. The second involved step is the edge detection, where the Canny edge detection method calculates the gradient using the derivative of a Gaussian filter and uses two thresholds to identify strong and weak edges [13,14]. With this approach, the detected edge pixels of unwanted noisy parts of the image are minimized. The edge is detected according to the function, *EM = edge* (*S*, *Canny*, *δ*, *σ***)**, where *S* denotes the pre-processed image, ‘Canny’ denotes the edge detection algorithm, δ = 0.4→0.02 is the hysteresis thresholding values used, *σ = sqrt* (2) is the standard deviation of the Gaussian filter and is a scalar, and EM is a binary matrix with 1 s representing the points where an edge is detected. The outcome of this process is presented in Figure 2b,c.

The next step of the process is the detection of the wall edges. We apply the well-known Hough Transform method [15] to detect the most prominent straight lines of edge pixels in the image. Since we are only looking for horizontal (ceiling and floor) and vertical (wall ends) lines in the image, we limit our line orientation search space to within +/−5° of 0° and 90°. We assume that the wall edges are the most prominent lines near the top, bottom, left, and right of the image. This method is robust to occlusions from furniture, as not the whole edge of the wall needs to be visible for a line to be detected. As long as part of the edge is visible, a line will be detected. The main challenge here is to keep only the four lines that correspond to the actual wall edges and discard other possible candidates. Non-occluded edges are easy to be detected, as they produce the strongest responses in the Hough Transform algorithm. The edge between the ceiling and the wall is usually fully visible and easy to be detected. The ceiling edge, as well as the ceiling corners detected, is used to facilitate the search for the other edges and eliminate false candidates. If no other edges can be detected, the ceiling edge and corners, together with the wall height parameter provided by the user, are used to estimate all other corners and edges.

The next step of the process is the wall corner detection. Corners are considered as the intersections of the prominent lines detected. To further improve the robustness of the system, we apply a corner detection step on the original image, and we compare the detected corners with the corner locations indicated by the line intersections. If the two agree to within a certain error tolerance, we assume that the detection was correct. Otherwise, we prompt the user to take another picture. The image with the detected edges and corners is also displayed to the user for verification. In the next paragraph, we explain our approach for corner detection.

The corner detection is the third step of the process, where the edge map of the image is processed to identify the candidate corners. The function *Corner = detectMinEigenFeatures* (*EM*, *q*, *G*) was used, where *EM* denotes the edge map, *q* is a scalar value between [0, 1] and denotes the corner strength and quality [13]. Parameter *G* is the Gaussian filter dimension, and it is an odd integer value in the range 3 to infinity. In our experiments, we set *G* = 3, as it was empirically determined to provide the most satisfactory results The Gaussian filter is used to smooth the gradient of the input image. To eliminate false corner points, the corner points from the top part of the wall that have approximately the same value y_i_~y_j_ are preferred, where *i* and *j* are the two candidate corner points from the top left and top right part of the wall. The overall concept is showcased in Figure 2. In addition, the corner points, from the top-left and bottom-left part of the wall, that have the same *x* value x_i_–x_m_ are preferred, where *i* and *m* are the two candidate corner points from the top and bottom left part of the wall. The final corner points used are those that provide the minimum Euclidean distance from the theoretical horizontal line that connects their center of gravity. For adjacent walls that form a corner, facets should be grouped together to form a vertical edge. In case the corner detection process resulted in a slight mismatch on the corners of the vertical edge from the adjacent walls, a corner correction process was used to connect the two corner coordinates in their center of gravity forming a joint corner. 

We compute the dimensions of the wall according to the number of pixels between the top corner points and the height of the wall such as $w=r_{p}\cdot \left|x_{i^{*}}-x_{j^{*}}\right|$, where rp=h|yi*−ym*| is the pixel resolution measured in meters per pixel. The height of the wall, *h*, is an input that is provided by the user. Assuming that the camera position, when the picture was taken, was approximately equal from all edges, i.e., the camera was facing toward the center of the wall, the length of the horizontal lines can be estimated based on the number of pixels between corners.

Combining the four separate two-dimensional wall planes of a room into a 3D parallelepiped model is a straightforward process, assuming that the user took the photos in a clockwise manner. The Geomagnetic Rotation Vector provided by the Android API can also be used to verify the direction of the camera when each picture was taken and warn the user if an inconsistency is detected.

Door detection is a slightly less complicated process due to the standardized dimensions of the door heights and widths. The door detection process follows a similar approach, where an edge and corner detection algorithm is used to find the location of the boundaries of the door. To increase the efficiency of the door corner detection algorithm, the following conditions were assumed: (a) preferred door corner should have a *y*-axis value relatively equal to the standard door height of 2.1 m, (b) two corner points should have relatively the same *y*-axis values, (c) two corner points should be separated relatively by standard door width 0.9 m.

### 3.2. Min-Max Spatial Mapping

**Figure 3:**
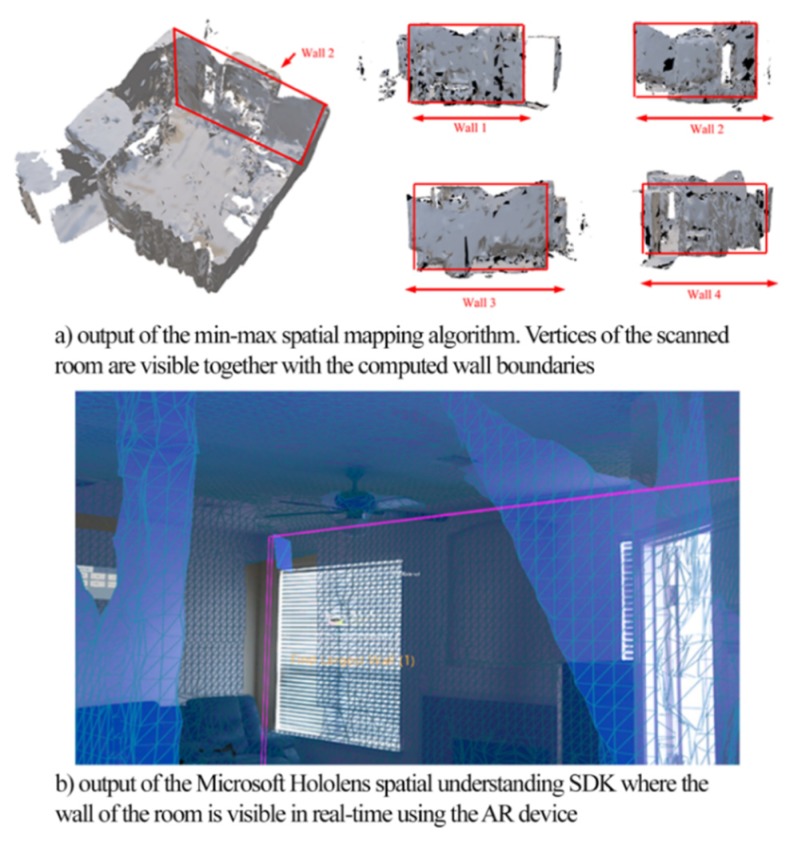
Outputs of Min-Max and Microsoft SDK spatial understanding algorithms.

This algorithm processes the object file derived by the spatial mapping process of Microsoft HoloLens and creates the facet model of the indoor environment suitable for ray-tracing simulations. With the use of the AR device, the user is required to scan the environment of interest. The depth cameras perform the process called spatial mapping, and the outcome of this process is an .obj file that contains a large number of vertices, similar to the concept of point cloud. The spatial map data will consist of three main pieces of information, i.e., vertices, vertices normal, and fragments. The Microsoft HoloLens maps the data in terms of small triangles where the vertices represent each point of a triangle. Vertices normal represent the direction of the object or the wall direction. Fragments data represent the graphical representation of the spatial map. An illustration is given in Figure 3a. 

The proposed min-max spatial mapping algorithm incorporates three main steps, (a) create clusters of vertices from the same walls of the room, (b) identify the corner vertices for each wall and (c) calculate the dimensions of the walls of the room. Once this is performed, the facet model of the room can be computed. For the clustering phase, we use the normal of the vertices as the key variable. By considering the illustration of the indoor room presented in Figure 3a, we identify the following clusters of vertices. Wall_1 incorporates all vertices with X positive normal direction, wall_2 incorporates all vertices with Z positive normal direction, the cluster of wall_3 incorporates all vertices with an X negative normal direction, and the cluster of wall_4 incorporates all vertices with Z negative normal direction. Thus, four different vertices groups are available.

Once the clusters of vertices are identified, the corners of each wall are computed using the proposed min-max concept. For each cluster, the vertices with the maximum and minimum values of the *x*, *y*, *z*-axes were filtered as the wall corner points. The dimension of each wall will be the distance between the two extreme vertices’ points. The length, height, and breadth of a room was computed according to the difference of the min and max values of their coordinate *x*, *y*, *z* values. This algorithm does not provide an accurate reconstruction of the environment since it is not capable of identifying doors and windows that may significantly change the signal variation. For the purpose of our investigation, the Min-Max algorithm was only used for testing of the provided accuracy and as the simplest in terms of computational needs solution. 

### 3.3. Hololens SDK Spatial Understanding

This approach utilizes an existing Software Development Kit (SDK) of Microsoft Hololens, which is capable of recognizing surfaces such as floors, walls, and ceilings [14]. The SDK is an inbuilt Hololens set of AI and object detection functions that utilize image processing and spatial mapping solutions. Within the Dynamic Link Library (DLL), the topology manager handles the labeling of the environment. Heuristics are used for determining the floor, ceiling, and walls. For example, the largest and lowest horizontal surface with greater than 1 m^2^ surface area may be considered as the floor [14]. A subset of the queries exposed by the Topology manager is exposed out through the DLL. For the purpose of our investigation, we mainly used the function called *QueryTopology FindPositionsOnWalls* that provides information about the position of the walls. The structure *TopologyResult* provides the numbered values of the results, which is then used to compute the facet model. The used code snippet is given in Algorithm 2 that presents the code snippet. The outcome of the process is shown in Figure 3b. Similar to the Min-Max algorithm, this technique was also used for comparison purposes.
**Algorithm 2.** Capturing Wall Dimensions from Hololens SDKEXTERN_C__declspec(dllexport) int QueryTopology_FindPositionsOnWalls(_In_ float minHeightOfWallSpace,_In_ float minWidthOfWallSpace,_In_ float minHeightAboveFloor,_In_ float minFacingClearance,_In_ int locationCount,_Inout_ Dll_Interface::TopologyResult* locationData)---------------------------------------------------------------------------------struct TopologyResult {DirectX::XMFLOAT3 position; DirectX::XMFLOAT3 normal; float width;float length;};

## 4. Constructing the Environment and Algorithm Limitations

**Figure 4:**
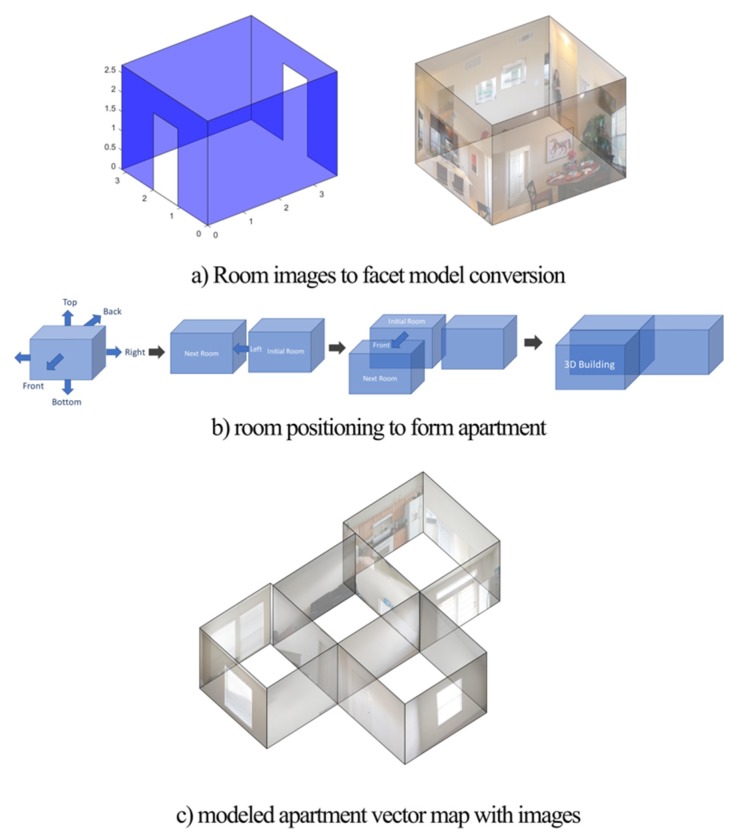
Indoor reconstruction steps.

Each facet represents an individual wall described by the four corner coordinates. Following a similar approach, the facet model of all remaining rooms is computed, and combined to form the vector map of the indoor environment. We assume that the user scans each room to cover the 360 space in a clockwise manner. Once the user completes this process for one room, then the user scans the adjacent room, starting from the wall that is shared with the previous room. In that way, there is always a reference point that allows the algorithm to reconstruct and attach the facet of each room in a realistic way to reconstruct the indoor environment. The output of this process is presented in Figure 4. The facet model incorporates data in a structured manner such as room(i).facet(j).coordinates, which describes the coordinates of the facet as a set of four corners, room(i).facet(j).material that describes if the material of a facet is brick or concrete to represent a wall or wood for doors or glass for windows. Finally, the room(i).facet(j).type structure that describes if the facet is a wall, door or window, and room(i).facet(j).width represents the width of the material. For the purpose of our investigation, the constitutive parameters and types of used materials in Table 1. 

The proposed algorithm is a first approach to tackle the problem of in situ network visualization using AR and ray-tracing algorithms and presents some important limitations. The most important are summarized as:

- it can be used only for simple residential apartments and cannot provide accurate results for large multi-story homes or commercial buildings due to the complexity of the clutter of the indoor environment,
- does not consider furniture clutter that may be important for mm-Wave propagation,
- the algorithm requires that images should be aligned and not rotated and taken in a clock-wise manner,
- large corridors cannot be efficiently captured in the images.

Furniture was not modeled and not considered in the ray tracing algorithm since, at the frequency range of 6 GHz, it does not create significant field variations.

One of the challenges of mm-Wave propagation is that medium and small size objects, such as furniture, may significantly affect signal strength. The incorporation of furniture data in the vector indoor map is a challenging task due to the complex geometrical structures of furniture. In addition, the small size of furniture surfaces may be comparable to the wavelength of mm-Wave waves, and ray tracing and Geometric Optics (GO) approximations may not be valid. In that case, full wave propagation approximations may be necessary for radio predictions that will significantly increase the complexity of the solution and potentially degrade the User Experience (UX). An alternative solution may be the use of empirical models that account for furniture shadowing [16], but this is out of the scope of this investigation. In addition, existing image recognition tools are able to recognize furniture objects but they cannot geometrically model them with the facet approximation. To that end, the incorporation of mm-Wave propagation and furniture in the indoor reconstruction algorithms will be investigated in a future version of this research work. Finally, the presented indoor reconstruction algorithms cannot deal with large and complex environments. One potential way to deal with this issue is to use point cloud data from laser scanners. This approach will be included in the future work of this research and is out of the scope of the current study.

## 5. The Ray Tracing Algorithm

The ray-tracing engine performs the theoretical predictions of the field strength over the scanned environment. The complex indoor scenario imposes clutter between the transmitter and the receiver, causing rapid field variations. For the purpose of our investigation, a ray-launching (shooting and bouncing) algorithm is developed that predicts field values as a combination of multiple reflections, multiple transmissions through walls, or a combination of both and a single corner diffraction [2], following principles from Geometric Optics (GO) and Uniform Theory of Diffraction (UTD). The input of the ray tracing engine is the facet model of the indoor environment, provided by the proposed spatial map to facet translation algorithms, and the output is a mesh grid where each element represents the predicted received signal strength for each coordinate point. The field at a given receiving position that consists of *N* ray paths is computed according to [2]:

(1) $$E=\overset{N}{\underset{i=1}{\sum }}E_{0}A_{i}\left(\overset{N_{Ri}}{\underset{j=1}{\prod }}R_{ij}\overset{N_{Ti}}{\underset{k=1}{\prod }}T_{ik}\right)e^{-ikr_{i}}+\overset{N}{\underset{i=1}{\sum }}UE_{0}A_{d}D_{i}e^{-ikr_{i}}.$$

In the above formulation, each ray path *i* can consist of *N_Ri_* reflections, *N_Ti_* transmissions, and a single diffraction. *R_ij_* and *T_ik_* are the air–wall–air interface reflection and transmission coefficients, and *D_ij_* is the wedge uniform theory of diffraction coefficient. The total path length is *r_i_*, and *A_i_*, *A_d_* represent the spreading factors of spherical waves and diffracted waves, respectively. *E*_0_ is the reference field at the transmitter. In addition, *k* is the wavenumber, *k* = 2*π/λ,* and U is an ON–OFF parameter, U = {0, 1}, indicating if diffraction occurs. The algorithm considered up to *N =* 5 interactions of the propagating rays with the indoor facets that could be reflections, transmission through walls, or any combination of the above. For complexity purposes, only single diffraction was considered. In [2], it is shown that these settings do not significantly affect the accuracy that is compared to real indoor measurements. The measurements were taken in an indoor environment that captured two scenarios as shown in [2]. In the first scenario, the transmitter was placed in the middle of a corridor and the receiver moved from a Line of Sight (LOS) to a Non Line of Sight (NLOS) region. This scenario was used to investigate the performance of the algorithm and its ability to compute field strength in free space and within multiple reflected propagation conditions (LOS) as well as diffracted and multiple transmitted-reflected conditions (NLOS). In the second measurement scenario, the receiver and transmitter were always in LOS inside a corridor and the receiver moved away from the transmitter. This scenario was used to investigate the performance of the algorithm and its ability to account for multiple reflections and path loss computations in free space. 

The air–wall–air interface model was used for the modeling of the reflection and transmission coefficients that considers the thickness of the clutter. The reflection coefficient was computed according to:

(2) $$R=\frac{1-e^{-j2\delta }}{1-R^{\prime 2}e^{-j2\delta }}R\prime ,$$

where

(3) $$\delta =\frac{2\pi d}{\lambda }\sqrt{\epsilon_{c}-sin^{2}\vartheta }.$$

For perpendicular polarization, the coefficient is:

(4) $$R^{\prime }=R_{p}=\frac{cos\theta -\sqrt{\epsilon_{c}-sin^{2}\vartheta }}{cos\theta -\sqrt{\epsilon_{c}-sin^{2}\vartheta }}.$$

The transmitted through walls field was computed based on:

(5) $$T=\frac{\left(1-R^{\prime 2}\right)-e^{-j\left(\delta -kd\right)}}{1-R^{\prime 2}e^{-j2\delta }}.$$

In the above formulations, *d* is the material thickness, *θ* is the angle of incidence, *k* is the wavenumber, *λ* is the wavelength, and the *ε_c_* is the complex permittivity that contains the relative permittivity value and the conductivity of the material as shown in Table 1 [17]. 

The diffraction mechanism was modeled according to a single Uniform Theory of Diffraction (UTD) Knife-edge diffraction:

(6) $$D=\frac{e^{-j\pi /4}}{2\sqrt{2\pi k}\cos\left(a/2\right)}F\left[2kLcos^{2}\left(a/2\right)\right],$$

where *F* is the transition function that incorporates Fresnel integrals, *L* is the distance parameter, and *a* is the diffraction angle [17].

The output of the ray tracing engine was delivered to the AR application that was responsible for overlaying the predicted channel estimation on the physical environment in the form of a holographic *colormap* and using the occlusion technique to provide a MR UX.

## 6. Results and Comparison

### 6.1. Comparison of Indoor Reconstruction 

**Figure 5:**
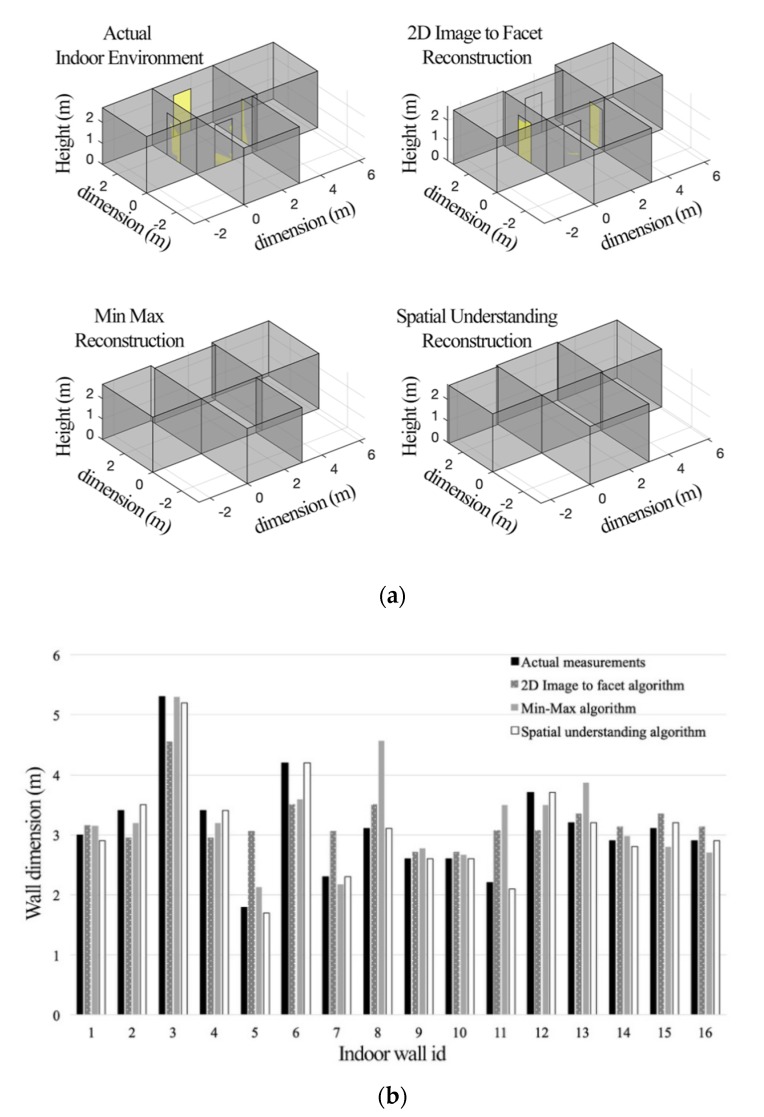
Indoor reconstruction from real measurements and the three spatial to facet model algorithms and comparison of the outcomes of the processes. (**a**) Visualization of the dimensions of the actual and reconstructed indoor environments; (**b**) quantitative comparison of the actual and estimated dimensions of each wall.

The proposed spatial map-to-facet translation algorithms were tested in a 2-bedroom residential apartment. The actual CAD file describing the geometry of the indoor environment was used as a reference to measure the accuracy of the performance of the developed algorithms. The results are presented in Table 2 and Figure 5. The figure presents the comparison of the performance of the three spatial map-to-facet translation algorithms in terms of the computed wall dimensions. We can see that they are very close to the actual wall dimensions, and it was found that the SDK spatial understanding algorithm performed better in terms of accuracy. This is also shown in Table 2. This is expected since this algorithm incorporates inbuilt AI engines in the Hololens AR device. On the other hand, the proposed low-cost 2D image-to-facet model algorithm, which does not require the use of depth cameras and AR devices, but rather a simple smartphone device, also performs acceptably. The accuracy of Table 2 was computed according to:

(7) $$e=\frac{\sum_{i=1}^{N}\sqrt{{\left(x_{i}^{m}-x_{i}^{a}\right)}^{2}+{\left(y_{i}^{m}-y_{i}^{a}\right)}^{2}+{\left(z_{i}^{m}-z_{i}^{a}\right)}^{2}}}{N},$$

where (*x,y,z*)_i_ represents the coordinate of the wall I and (*x,y,z*)^a,m^ represents the theoretical (a) and measured (*m*) position of the wall based on the real indoor CAD file.

### 6.2. Comparison of Field Predictions 

**Figure 6:**
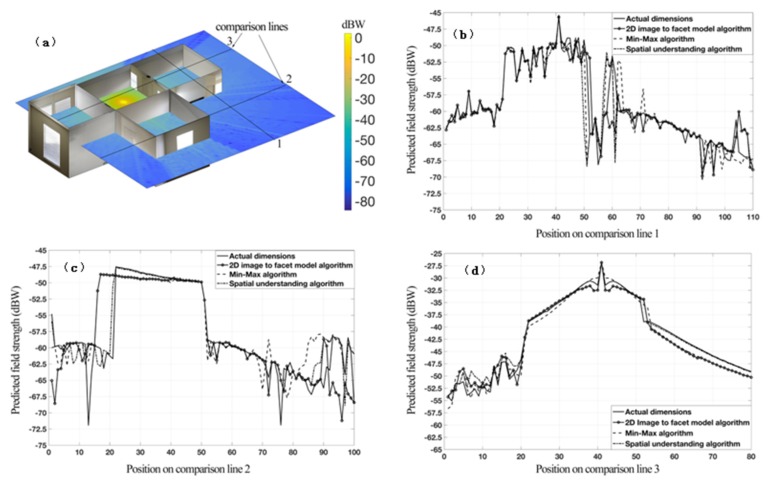
Field strength predictions as a 2D mesh grid and over the comparison lines. The comparisons present the differences in field predictions from the reconstructed indoor environments: **(a)** presents the field predictions over a 2D plane at 1.5 m above ground. (**b**) presents the predictions over the path line 1 whereas (**c**) and (**d**) present the predictions over the path lines 2 and 3 respectively.

The purpose of this section is to investigate the variations of the predicted field strength, considering the inaccuracies of the indoor reconstruction. The results are shown in Figure 6. The top-left part of the figure presents the signal strength predictions of the ray tracing algorithm. The signal strength is presented in dBW scale for a 6 GHz wireless signal. The transmitter was placed in the middle of the room for visualization purposes. The transmitter assumed to have a 0 dBd (dipole) gain and an initial transmit power of E_0_ = 0.1 W, which is the typical picocell power level. The interaction of the wireless signal with the clutter, walls or doors is visible in the surface plot (top-left image). In addition, the ray-tracing predictions over various comparison lines are plotted for each reconstruction algorithm, and compared against the measured field strength. The solid line represents the measured signal strength along the comparison line and the other three represent the predictions from the indoor environment as reconstructed by the 2D image to facet, Min-Max, and spatial understanding algorithms. We observe that the signal strength predictions are not significantly affected by the errors generated by the proposed spatial map to facet model translation algorithms, indicating that indoor reconstruction solutions such as the one presented in this paper are robust enough to be used for radio planning purposes using user-friendly AR applications. 

To further investigate the accuracy of between the field predictions, we calculated the Pearson correlation *r* between the measured signal strength and the corresponding predicted values at each measuring point, along the three comparison lines [18]. Assuming the variable *x* represents the measured values and *y* represents the predicted values, *r* is calculated as:

(8) $$r=\frac{SS_{xy}}{\sqrt{SS_{x}SS_{y}}},$$

where $SS_{x}=\sum {\left(x-\bar{x}\right)}^{2}$, $SS_{y}=\sum {\left(y-\bar{y}\right)}^{2}$ and $SS_{xy}=\sum \left(x-\bar{x}\right)\left(y-\bar{y}\right)$. Values of *r* close to 1 indicate a strong positive correlation, or, in other words, an accurate prediction, whereas values close to 0 indicate a weak correlation between the two variables, *x* and *y*. A total of 111 measured and predicted values were used for comparison lines 1 and 3, and 101 values for line 2. Note that the signal strength in Watts was used for calculating the correlation values, instead of dBW, in order to preserve the linear relationship in signal strength variations between the measured and predicted values. Table 3 below presents the Pearson correlation values for each method and each measurement line.

It is found that the differences between the predictions over the three reconstructed indoor environments are very correlated, and there should not be an important statistical difference between them. This makes the use of the proposed solution suitable for radio planning where the used radio propagation predictions should be close to the real values, maintaining the statistics of the signal variation. 

### 6.3. Real-Time Network Visualization

**Figure 7:**
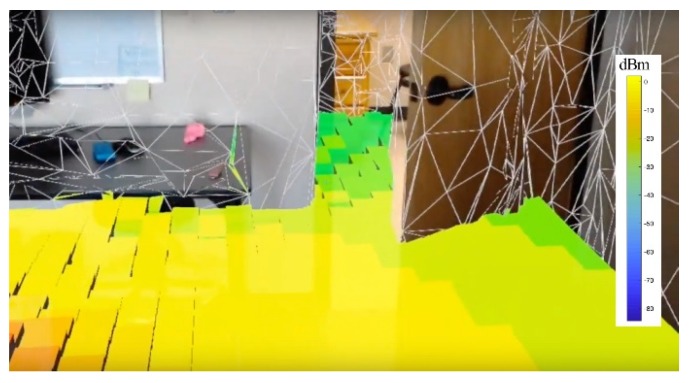
AR in situ network visualization. The user is wearing an AR headset and experiences real-time field strength visualization in the form of holograms. The occlusion effect is also presented at the door.

To leverage the features of the AR devices, we performed a real-time network visualization test, as presented in Figure 7. The user wore an AR headset and walked inside the lab. A holographic representation of the estimated field strength was available to the user who could now better visualize coverage issues and plan the femtocell network in a more efficient way. The spatial mapping object file of the lab was converted to a facet model, and this was used by the ray tracing algorithm. To minimize the time for the computation of the ray-tracing results, we reduced the number of ray interactions to 3 (instead of 5) and a grid resolution of 25 cm (instead of 10 cm). This provided fast results that can enable an acceptable UX. The ray-tracing results were then incorporated in the AR device that overlaid the colormap on the physical space. The feature of occlusion was used to provide a more realistic experience to the user, called MR. The overall time required to convert the spatial map data to ray tracing results depends on the grid resolution and the number of ray interactions with the environment. For this demo, the total execution time of all algorithms was 10 s. This means that the user is required to walk in the environment, scan the environment, and wait for 10 s for the holographic field predictions to be visualized and interact with the environment.

### 6.4. Complexity Analysis

This section provides an overview of the complexity of the proposed solution. The complexity analysis is divided into two sections. The first section describes the complexity of the image to facet (indoor reconstruction) algorithm that involves edge detection and image processing techniques. The second section describes the complexity of the ray tracing algorithm that involves traces of multiple rays in the space. 

The image to facet algorithm is responsible for processing an image and creating an indoor vector map based on the facet model. This process involves steps with different orders of computational complexities. The involved steps are presented in Algorithm 1 and the two most computational demanding processes are the Canny Edge Detection and the Hough Transform. In general, Canny Edge detection involves the following steps for every image. A convolution of the image with a blur kernel, four convolutions with edge detector kernels, the computation of the gradient direction, a non-maximum suppression, and thresholding with hysteresis. To implement convolutions using Fast Fourier Transform (FFT), the complexity in time is O(nlog(n)), where *n* is the number of elements of the image. If the image has dimensions *m* × *n* pixels, the estimated complexity will be O(mnlog(mn)). For the Hough Transform, the complexity increases at a rate of $O\left(P^{q-2}\right)$ with each additional parameter that is introduced, where *P = m × n* is the size of the image and *q* is the number of parameters. For the purpose of our investigation, the implementation of the Hough transform was focused on the detection of a straight-line existence. For the ray tracing algorithm presented in Section 5, the number of traced rays between two points is $R=\overset{N}{\underset{k=1}{\sum }}F{\left(F-1\right)}^{k-1}$, where N is the number of interactions and F is the number of facets in the environment [19]. The computation time increases at a rate of $O\left(R\cdot F^{N+1}\right)$. 

## 7. Conclusions

This paper presented a novel network coverage visualization technique that integrates ray tracing, AR and machine learning technologies such as Spatial Understanding SDK algorithms of existing AR devices. The proposed system model converts spatial map data from an AR device or a phone camera to a facet model, suitable for ray tracing field predictions. A ray tracing method was implemented, based on the ray launching principle, to provide field strength predictions over the scanned environment. This data was then integrated into an AR application that overlaid a holographic presentation of the field strength in the indoor environment. With the expected densely deployed 5G networks and the evolution of AR and MR technologies, we believe that this type of user-friendly applications will improve the network planning process. In the future work of this research, we plan to productize the developed solution and evaluate the efficiency of indoor networking planning with users that are not experts in the field. Finally, the proposed system can also be used for educational purposes to teach undergrad and graduate students the basics of radio propagation and channel estimation. This will be explored in our future work.

## Figures and Tables

**Table 1:** Constitutive parameters of used materials.

Material	ε_r_ (F/m)	σ (S/m)	Thickness (cm)
Brick Wall	4.4	18 × 10^−3^	15
Wood door	1.9	8 × 10^−3^	5
Window	5.2	3.5 × 10^−3^	1

**Table 2:** Comparison of spatial map to facet translation algorithms.

Algorithm	Accuracy
Image to facet algorithm	93%
Min-Max algorithm	95.6%
Spatial understanding SDK	96.6%

**Table 3:** Pearson correlation coefficient between the measured signal strength and the predicted strength by each algorithm.

	Algorithm	Correlation (*r)*
Comparison Line 1	Image to facet algorithm	0.9084
	Min-Max algorithm	0.9403
	Spatial understanding SDK	0.9308
Comparison Line 2	Image to facet algorithm	0.8595
	Min-Max algorithm	0.8581
	Spatial understanding SDK	0.9877
Comparison Line 3	Image to facet algorithm	0.9885
	Min-Max algorithm	0.9897
	Spatial understanding SDK	0.9977
